# 

**Published:** 1917-12-22

**Authors:** 


**Rates of Subscription:** Twelve months, 6/6; Six months, 4/-; three months, 2/-, post free to any address in the United Kingdom. Abroad, Twelve months, 8/8; Six months, 5/-; Three months, 2/6, post free. THE HOSPITAL "Index" to Volume LXII., April 1917 to September 1917, is now ready, price 2½d. per copy, post free. Address The Manager, THE HOSPITAL, "The Hospital" Building, 28/29 Southampton Street, Strand, London, W.C. 2.

### Leprosy.

In the House of Commons, last week, Colonel Yate asked the President of the Local Government Board how many cases of leprosy there are in this country; and whether steps are being taken to segregate persons suffering from leprosy and to provide for their treatment and maintenance. Mr. Walsh replied that there is no exact information, but the President is informed that there are only a few cases in the country, and that the disease is not spreading here. The infectivity of the disease is very limited, and measures for segregation would not be justified at present. Valuable work in connection with such cases is being done by private funds.

### Matrons' and Sisters' Appointments.

DYKEBAR WAR HOSPITAL, PAISLEY.—Miss Marion P. Jeffrey has been appointed night matron. She was trained at the Western Infirmary, Glasgow, and at Bangour Village Asylum.

ISOLATION HOSPITAL, SPENNYMOOR.—Miss Ida Ingram has been appointed nurse-matron. She was trained at Mile End Old Town Infirmary, and has been matron at Borough Isolation Hospital, South Shields.

AUXILIARY MILITARY HOSPITAL, ARDMILLAN, OSWESTRY.—Mrs. Muriel O. Hughes has been appointed sister-in-charge. She was trained at the London Hospital, has been nursing sister under Queen Alexandra's Royal Naval Nursing Service Reserve at Haslar and the Royal Naval College, Osborne, I. of W., day sister at Sir Charles Henry's Hospital, Parkwood, Henley-on-Thames, and night sister at the Red Cross Hospital, Manor Hill, Birkenhead.

BLEASDALE HOUSE AUXILIARY HOSPITAL.—Miss Caroline Clara Boness has been appointed sister. She was trained at the Anlaby Road Infirmary, Hull, has been charge nurse at the Infirmary, Old Windsor, sister at No. 10 B.R.C. Hospital, France, and staff nurse at King George Hospital, London.

CITY HOSPITAL FOR INFECTIOUS DISEASES, NEWCASTLE-ON-TYNE.—Miss F. M. Rothera has been appointed ward sister. She was trained at the Royal Halifax Infirmary, and at the Kendray Hospital, Barnsley.

**NOTE.—Particulars of Appointments for insertion in this column will be welcomed.**

### Held Over.

WE much regret that great pressure on our space compels us to hold over an interesting article on the Commemoration of the First Seven Divisions at the Albert Hall, some College news, the *Institutional Worker*, and other items.

